# Crystallization Kinetics Analysis of the Binary Amorphous Mg_72_Zn_28_ Alloy

**DOI:** 10.3390/ma16072727

**Published:** 2023-03-29

**Authors:** Bartosz Opitek, Beata Gracz, Janusz Lelito, Witold K. Krajewski, Mariusz Łucarz, Piotr Bała, Tomasz Kozieł, Łukasz Gondek, Michał Szucki

**Affiliations:** 1 Faculty of Foundry Engineering, AGH University of Science and Technology, 30 Mickiewicza Street, 30-059 Cracow, Poland; 2 Faculty of Metals Engineering and Industrial Computer Science, AGH University of Science and Technology, 30 Mickiewicza Street, 30-059 Cracow, Poland; 3 Faculty of Physics and Applied Computer Science, AGH University of Science and Technology, 30 Mickiewicza Street, 30-059 Cracow, Poland; 4 Foundry Institute, Technische Universität Bergakademie Freiberg, 4 Bernhard-von-Cotta-Str., 09599 Freiberg, Germany

**Keywords:** amorphous MgZn alloy, metallic glasses, crystallization kinetics, thermal stability, Kissinger model

## Abstract

The aim of the study was to analyze the crystallization kinetics of the Mg_72_Zn_28_ metallic glass alloy. The crystallization kinetics of Mg_72_Zn_28_ metallic glass were investigated by differential scanning calorimetry and X-ray diffraction. The phases formed during the crystallization process were identified as α-Mg and complex Mg_12_Zn_13_ phases. Activation energies for the glass transition temperature, crystallization onset, and peak were calculated based on the Kissinger model. The activation energy calculated from the Kissinger model was *E*_g_ = 176.91, *E*_x_ = 124.26, *E*_p1_ = 117.49, and *E*_p2_ = 114.48 kJ mol^−1^, respectively.

## 1. Introduction

Magnesium alloys are characterized by low density and good mechanical properties, thanks to which castings from these alloys have been used in many industries, primarily in the aviation and automotive industries [1,2,3,4]. In addition to these advantages, magnesium alloys also exhibit good biocompatibility with the human body. This feature also makes magnesium alloys suitable for use in medicine [5,6,7,8,9,10]. Magnesium and its alloys, which are chemically active, may undergo natural degradation in physiological environments as a result of corrosion. Because of this property, they are potential materials for applications in biodegradable hard tissue implants. Biodegradable magnesium implants are intended to dissolve and then be excreted or absorbed by the human body after tissue healing [9,10]. This would mean a shortening of patients’ recovery time as well as a reduction in treatment costs. However, the excessive rate of degradation and the resulting evolution of hydrogen [11,12] has led to the abandonment of further work on the biomedical use of crystalline magnesium alloys. The literature can also be found with studies on improving the energy condition of metallic glasses leading to the formation of nanostructural metallic glasses, expanding their use, such as highly efficient catalysts for energy conversion [13].

Recent years have seen renewed interest in magnesium and its alloys as a biodegradable material for metal orthopedic implants. Research has shown that magnesium alloys can be used, but in the form of alloys with an amorphous structure containing magnesium (Mg), zinc (Zn), and calcium (Ca) [14]. It should be noted that these elements are non-toxic and also are present in the human body. Metallic glasses, due to their amorphous structure, characterized by the lack of grain boundaries, can provide a material with better mechanical properties and higher corrosion resistance than crystalline materials. Numerous studies of the kinetics of crystallization of amorphous ternary Mg-Zn-Ca and Ca-Mg-Zn alloys with different chemical compositions can be found in the literature [15,16,17,18,19,20]. Unfortunately, the widespread use of amorphous alloys is limited due to their low plasticity [21]. The process of partial crystallization of amorphous alloys may be a solution allowing plasticity to be improved with only a slight deterioration of corrosion resistance. However, there is little information in the literature on the kinetics of crystallization of two-component Mg-Zn amorphous alloys [22,23,24]. The authors of Calka et al. [22] conducted a detailed study of the crystallization kinetics of the amorphous Mg_70_Zn_30_ alloy. They observed three exothermic peaks, the first two occurring in the lower temperature range and the third in the higher temperature range. The first two overlapping exothermic peaks were related to crystallization, while a much smaller peak occurred at higher temperatures. X-ray and electron diffractograms showed the presence of only the Mg_7_Zn_3_ phase, called Mg_51_Zn_20_. They also found that these two overlapping exothermic peaks are not due to the sequential formation of two different structures. They also found that the first exothermic peak may be caused by the formation of very fine grains of the Mg_51_Zn_20_ phase, which are disordered in terms of composition. The second exothermic peak is related to the recrystallization process to just perfect grains and the crystallization of the remaining amorphous material. The weaker third exothermic peak is due to the precipitation of some magnesium from the Mg_51_Zn_20_ phase as well as grain growth. Boswell [23] researched the crystallization of the Mg_74_Zn_26_ amorphous alloy. The crystallization consisted of the formation of a fine-grained Mg_51_Zn_20_ phase and subsequent recrystallization of this phase. As a result, a more perfect Mg_51_Zn_20_ and α-Mg phase was obtained. Altounian et al. [24] noticed that the crystallization process of an amorphous Mg-Zn alloy is initiated at low temperatures by the precipitation of a free-grained, deformed Mg_51_Zn_20_ phase, which at the end of the crystallization process grows into regular Mg_51_Zn_20_ crystals. They also found that the α-Mg phase also precipitates in Mg-rich alloys. The mechanism of further recrystallization at higher temperatures depended on whether the alloy was rich in Mg or Zn in relation to Mg_51_Zn_20_.

Knowledge of the kinetics of crystallization of amorphous Mg-Zn alloys may provide information on the effect of other alloying elements, such as calcium, on the course of the crystallization process because amorphous alloys are metastable materials that crystallize with increasing temperature and thus transition to a stable state. Therefore, from the technological and utility point of view, the stability of amorphous alloys is of great importance. One of the kinetic parameters, which simultaneously proves the stability, is the activation energy. Therefore, conducting an analysis of the crystallization kinetics of two-component amorphous Mg-Zn alloys is justified.

## 2. Materials and Methods

In order to obtain the eutectic alloy Mg_72_Zn_28_, magnesium and zinc with a purity of 99.9% were used. The chemical composition of this alloy consisted of Zn: 28 at.% and Mg: 72 at.%. Subsequently, melting was carried out in a resistance furnace under an argon shield as an inert gas. After melting, the liquid alloy was cast into a steel mold to obtain a cylindrical sample of 20 mm diameter and 50 mm height. From the cylindrical sample cast in this way, a sample with a diameter of 10 mm was bored for further testing. The sample was re-melted using a melt spinning device to produce amorphous ribbons 150 μm thick. Both the melting and casting process took place under an argon shield. The circumferential speed of the wheel was 40 m/s. The kinetics of amorphous alloy crystallization was characterized by continuous heating in a TA DSC Q20 under high-purity argon flow. The heating rates were 5, 10, 20, 40, and 80 K/min. Studies have also been carried out using X-ray diffraction. X-ray diffraction was used to confirm the amorphous nature of the ribbons prepared in this way. X-ray diffraction was also combined with continuously heating the amorphous ribbon at 5 K/min. These studies identified the phase components formed during heating. Additional studies on the kinetics of amorphous alloy crystallization Mg_72_Zn_28_ were carried out using variable temperature diffraction (XRD). These tests were carried out at an isothermal annealing temperature of 352 K or 355 K. X-ray diffraction (XRD) test results were collected using a Panalytical Empyrean diffractometer equipped with a Cu Kα X-ray source. The non-ambient temperature studies were performed in an Anton Paar HTK 1200N chamber. The position of the sample was corrected for thermal displacement, and the temperature stabilization was better than 0.2 K.

Activation energy is one of the three kinetic parameters. Isothermal and non-isothermal methods can be used in calorimetric measurements. Most methods are based on the Kolmogorov-Johnson-Mehl-Avrami (KJMA) isothermal transformation kinetics equation [25,26,27,28,29]:

(1) $$X=1-\exp[-{\left\{K\left(t-\tau \right)\right\}}^{n}]$$

where *X*(*t*)—fraction of crystalline phase, *t*—crystallization time, *τ*—incubation time, which is associated with the delay of the beginning of the transformation, *n*—Avrami exponent, which is associated with the nucleation mechanism and growth geometry. The Avrami exponent *n* value ideally takes integer or half-integer values. In the case of three-dimensional (3D) growth and sporadic nucleation, the exponent value is *n* = 3 + 1. This usually applies to polymorphic and eutectic transformations. However, when the transformation consists only of 3D growth on previously existing nuclei present at the time of *t* = 0, then *n* = 3. When the exponent *n* = 2.5, the transformation proceeds with a constant rate of nucleation, and the growth is parabolic, i.e., controlled by diffusion, slowing down over time at a given temperature. Where the Avrami exponent assumes a value of *n* = 1.5, the transformation is based solely on diffusion-controlled growth from pre-existing nuclei. In Equation (1), there is also the reaction rate constant *K*. This constant depends on the nucleation rate and the crystal growth rate. Using the Arrhenius equation, the reaction rate constant can be calculated [30]:

(2) $$K=K_{0\;}\exp\left(-\frac{E_{a}}{RT}\right),$$

where *K*_0_ is the frequency factor, *E*_a_ is the activation energy, and *R* is the gas constant.

Because the non-isothermal transformation is associated with a constant heating rate, the relationship between the temperature *T* and the heating rate *β* can be described by a linear relationship:

(3) $$T=\beta t+T_{0}$$

The Kissinger method developed in 1957 is based on the KJMA model and the logarithmic form of Equation (2) and is described by

(4) $$\ln\left(\frac{\beta }{T^{2}}\right)=-\frac{E_{a}}{RT}+const$$

The Kissinger method is used to determine the activation energy. Plotting a plot of ln (*b*/*T*^2^) depending on (1000/*T*), a straight line is obtained, from which slope *E*_a_ is calculated.

## 3. Results

**Figure 1:**
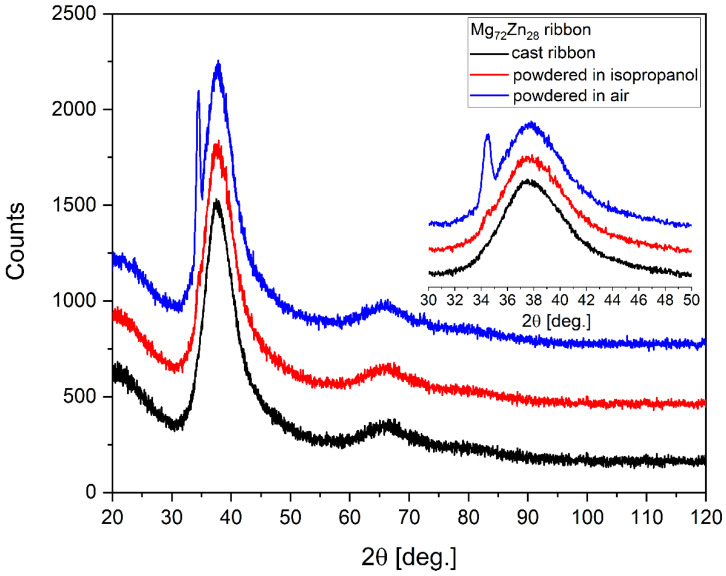
The X-ray (Cu Kα) diffraction patterns of Mg_72_Zn_28_ powdered and cast ribbon.

In order to check the amorphicity of the structure of the ribbon cast from the Mg_72_Zn_28_ alloy, these were subjected to XRD tests. The XRD pattern of the ribbon is shown in Figure 1. The ribbon powdered using agate mortar (in air) showed a reflection of α-Mg over the broad, amorphous feature. After milling the ribbon in isopropanol, the reflection almost vanished (see the insert in Figure 1). Therefore, it was shown that the alloy in the verge of crystallization due to the pressure/temperature raised during mechanical treatment. For the as-cast ribbon, the amorphous nature of the sample was evidenced.

**Figure 2:**
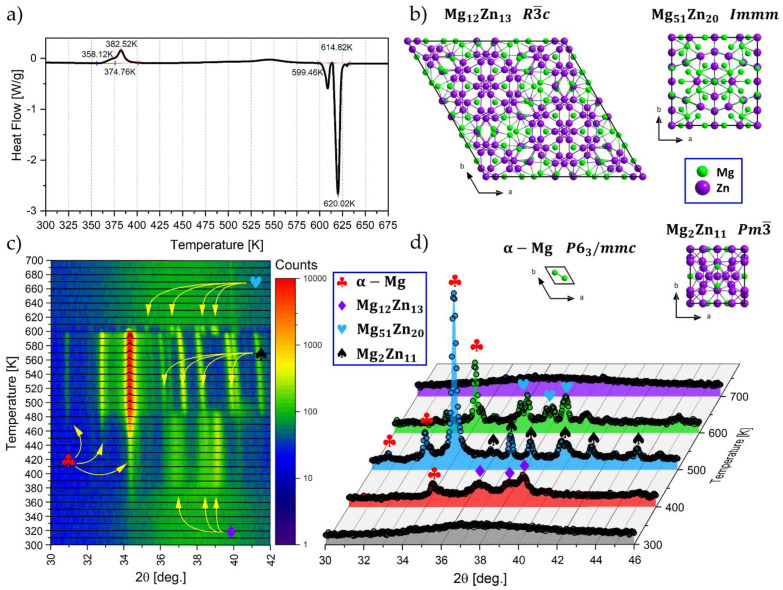
The course of the ribbon heating to 725 K with a rate of 5 K/min from DSC (**a**), recognized constituting phases (**b**), and high-temperature XRD studies (**c**,**d**).

In order to select the temperature of isothermal annealing, the amorphous ribbon was heated to a temperature of about 725 K at a speed of 5 K/min (Figure 2). The curve shows two overlapping exothermic peaks, one smaller exothermic peak, and two separate endothermic peaks. The first two overlapping exothermic peaks are related to the crystallization of two phases. At 358.12 K, the α-Mg phase crystallizes first (smaller peak), and then, at a temperature of about 374.76 K, the Mg_12_Zn_13_ phase crystallizes (bigger peak). Thus, in the temperature range between 358.12 K and 374.76 K, there are two structural components: amorphous and crystalline α-Mg phases. Two maxima can be distinguished for these two overlapping exothermic peaks, indicating the maximum heat release intensity. The first is at a temperature value of 371.0 K (α-Mg), and the second is at 382.52 K (Mg_12_Zn_13_). A small exothermic peak is visible in the temperature range from 475 K to 599 K and is associated with the Mg_2_Zn_11_ crystallization procedure. The first endothermic peak relates to the eutectic transformation, and the second to the melting of α-Mg. This allowed us to identify the initial crystallization temperature (*T*_x_ = 358.12 K) and the eutectic temperature (T = 614 K). The total amount of heat obtained from exothermic peaks is ΔH_exo_ = 55 J/g, while from endothermic peaks is ΔH_endo_ = 191 J/g. Thus ΔH_exo_ is about three times smaller than ΔH_endo_. The same phenomenon was observed by the authors of the publication [22]. This phenomenon is probably due to the large excess specific heat of the supercooled liquid relative to the solid and perhaps a small loss of enthalpy during heating to the crystallization temperature. The very low temperature of the beginning of crystallization of the two-component Mg-Zn alloy compared to the three-component Mg-Zn-Ca alloy (about 506 K) [15,16] suggests the low stability of the amorphous structure of the two-component alloy at ambient temperature. This may lead to spontaneous crystallization of this alloy at ambient temperature. The value of the initial crystallization temperature allowed two values of the isothermal annealing temperature (352 K and 355 K) to be selected, which are lower than the initial crystallization temperature.

In Figure 2b, the identified crystal phases during high-temperature XRD experiments are depicted. As apparent from Figure 2c,d, initially amorphous Mg_72_Zn_28_ alloy transforms at about 358 K into α-Mg and then at a temperature of about 374 K into the complex Mg_12_Zn_13_ phase. The latter phase is often described in the literature as ZnMg or Mg_21_Zn_25_. It is visible that peaks are not sharp, indicating a relatively low level of atomic order. In temperatures of 480–490 K, the reflections originating from α-Mg become well-developed, while reflections from the Mg_12_Zn_13_ phase are sharpened and shifted. The changes in intensities reflect a deficiency of Mg in the Mg_12_Zn_13_ unit cell due to the precipitation of α-Mg, as mentioned above. Above 490 K reflection originating from Mg_12_Zn_13_ disappeared, while the contribution of α-Mg became significantly higher. Simultaneously the Zn-rich Mg_2_Zn_11_ phase appeared. At rising temperatures, reflections originating from α-Mg move toward higher 2θ angles, indicating a decreasing unit cell volume. This is not surprising, as the solubility of Zn into α-Mg rises with temperature. Therefore, owing to Zn’s smaller metallic radius, the α-Mg lattice shrinks.

At the temperature of 600 K, the next transition takes place. The α-Mg and Mg_2_Zn_11_ phases gradually dissolve to create a magnesium-rich Mg_51_Zn_20_ alloy. At 610 K, the reflections from crystalline phases disappeared, confirming the achievement of reaching the melting temperature. The evidence sequence of phase transitions is in good agreement with the calculated and derived phase diagram of the Mg-Zn system [31]. The patterns at 700 K are typical of liquid metals, which are just short correlations giving glassy-like broad maxima. The patterns of the liquid phase are similar to the pattern of amorphous material, as expected.

To summarize, the emerging phase components during crystallization, caused by the heating of the alloy from the ambient temperature to the melting temperature, differ from those included in the publications [22,23,24]. This difference may result from the chemical composition of the tested alloy and the heating speed. The authors of the publication [22,23,24] conducted the research at the heating speed of 80 k/min, while the authors of this manuscript conducted the study at a speed of 5 K/min. A much lower heating rate causes approaching equilibrium conditions in which diffusion processes will play a dominant role.

**Figure 3:**
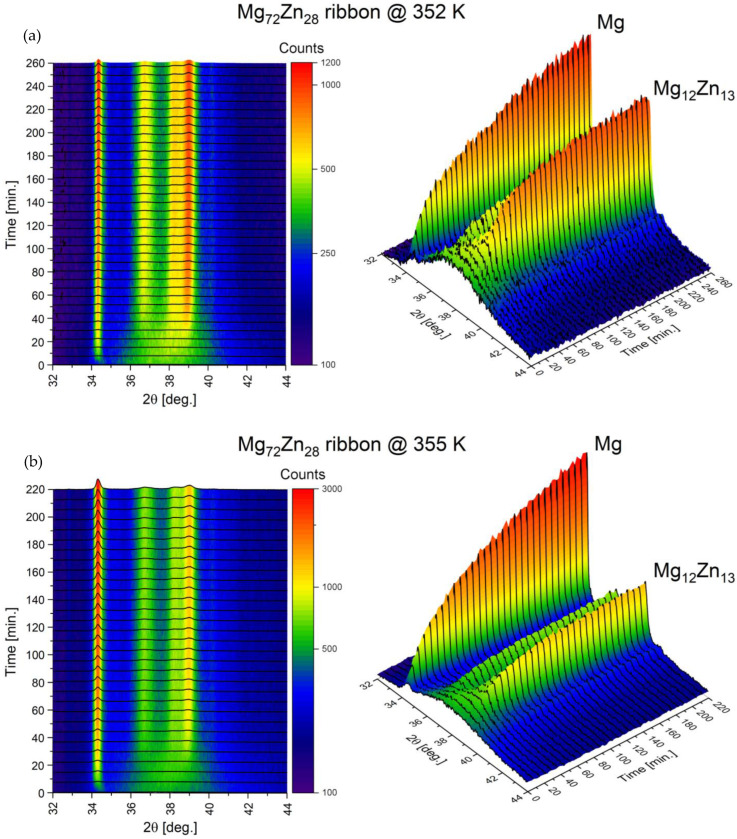
Evolution of XRD patterns as a function of annealing time at an isothermal annealing temperature of 352 K (**a**) and 355 K (**b**).

The results of the thermal and kinetic crystallization analysis of the amorphous Mg_72_Zn_28_ alloy for isothermal annealing temperatures of 352 and 355 K, carried out using the X-ray method, are shown in Figure 3. The proportion of the crystalline phase, both α-Mg and Mg_12_Zn_13_, increases exponentially and finally slows down, with only the Mg_12_Zn_13_ phase reaching a plateau in the time period studied. The α-Mg phase will probably also reach a plateau, requiring a longer annealing time. These studies confirmed the sigmoidal nature of the S-shaped phase change. In addition, these studies allowed the α-Mg phase and the Mg_12_Zn_13_ intermetallic phase to be identified as a phase formed during the crystallization process.

As evidenced, the crystallization strongly depends on temperature. For lower temperatures (352 K), the strongest reflections of α-Mg and Mg_12_Zn_13_ arise nearly to the same level, and the α-Mg phase is only slightly ahead of the Mg_12_Zn_13_ phase. At the same time, the diffusive background of the amorphous phase decreases; however, even after 260 min, it is still visible. On the other hand, at 355 K, the reflection of α-Mg grows much stronger. Moreover, the diffusive background decreases to a lower level compared to behavior evidenced at 352 K. This is a clear sign that the remaining amorphous contribution is constituted mainly by Mg. The achieved saturation of Mg_12_Zn_13_ reflections’ growth, in contrast to the continuous growth of reflection associated with α-Mg, supports this finding entirely.

**Figure 4:**
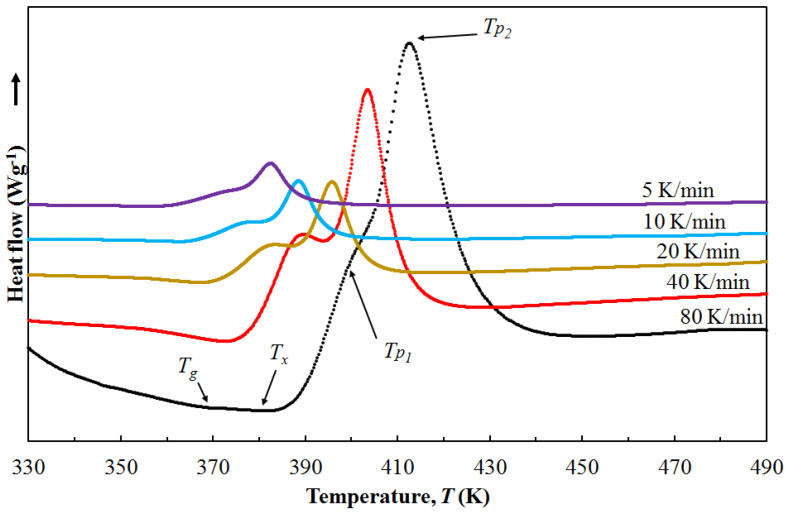
DSC curves for the amorphous Mg_72_Zn_28_ alloy heated at the rate of 5 K/min, 10 K/min, 20 K/min, 40 K/min, and 80 K/min.

The amorphous Mg_72_Zn_28_ ribbon was tested by DSC. The amorphous ribbon was heated at different rates to the melting point. The set heating rates were 5 K/min, 10 K/min, 20 K/min, 40 K/min, and 80 K/min, respectively. The results of the tests are presented in the form of graphs in Figure 4. The curves as a function of temperature presented in Figure 4 allowed us to determine the characteristic temperature values, such as the temperature of the material transition to a glassy state—(*T*_g_), the temperature of the beginning of crystallization—(*T*_x_), at which first crystals appear in the matrix of the amorphous alloy. The last important temperature point is the maximum crystallization peak—(*T*_p_), accompanied by the maximum intensity of heat release. The determined characteristic temperature values of the tested amorphous alloy are in the range of temperature values from 355 K to 415 K. In addition, the supercooling liquid region (Δ*T*_x_) was calculated, which is the difference between the crystallization start and glass transition temperatures. Metallic glasses with a large value of supercooled liquid region show high thermal stability and high resistance to crystallization. All data were collected and presented in Table 1.

Analyzing the curves in Figure 4, a clear shift of the peak with the increase in the heating rate towards higher temperature values can be seen. In addition, the size of the peak also increases with the increase in the heating rate. This phenomenon indicates the kinetic nature of the glass transition and crystallization. Furthermore, it can be seen that for all values of the heating rate, two peaks connected to each other are visible. This phenomenon is related to the nucleation and growth of two phases simultaneously. In addition, no *T*_g_ temperature was observed at low heating rates. It is related to the fact that better glass formers on heating show a glass transition and then a supercooled liquid region, and then they crystallize. For worse glass formers, the crystallization starts more easily and can obscure the glass transition. In that case, heating in DSC shows no glass transition. Whether the glass transition is seen on heating for any metallic glass could depend on the heating rate. Faster heating pushes the onset of crystallization to higher temperatures, making it more likely that the glass transition is revealed. Binary metallic glasses are, of course, not as good glass formers as multi-component glasses. Therefore, it is usual that they do not show a glass transition. However, that is unlikely to be universal. Some binary glasses can show a glass transition when heated fast enough. Therefore, the temperature *T*_g_ was determined by heating the amorphous ribbon at different rates. Then, at high heating rates, a change in the baseline slope was observed, which was attributed to the temperature *T*_g_. A slight increase in heat capacity was observed at *T*_g_.

**Figure 5:**
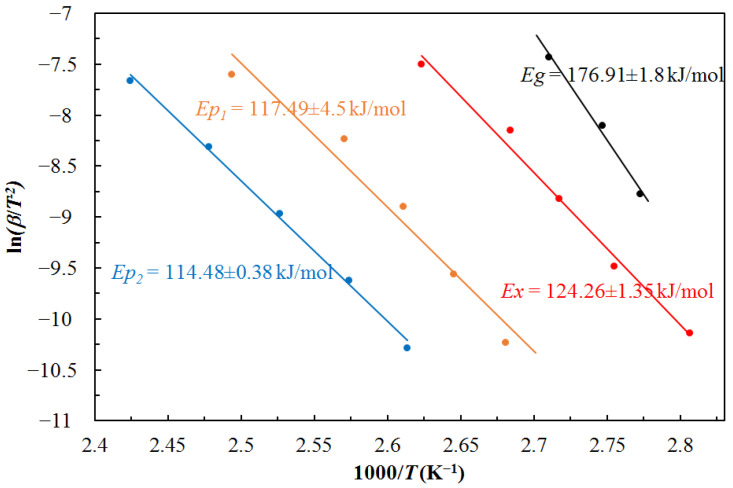
Activation energies for glass transition, crystallization start, and crystallization peak were calculated using the Kissinger method.

The activation energy was calculated to indicate the lowest possible energy value needed to carry out the appropriate reaction. Using the Kissinger method (Equation (4)), the activation energy was calculated for each characteristic temperature value of the Mg_72_Zn_28_ amorphous alloy, i.e., for the temperature of the glass transition, temperature of the crystallization and temperature for the maximum of the crystallization peaks [32]. The results of activation energy calculations are shown in Figure 5, respectively activation energy: *E*_g_ for the glass transition temperature, *E*_x_ for the crystallization temperature, and *E*_p1_ and *E*_p2_ for the temperature of the maximum of the peak crystallization, i.e., the maximum amount of heat released, respectively for the α-Mg phase and the Mg_12_Zn_13_ phase. The highest activation energy value was calculated for the glass transition temperature of the amorphous Mg_72_Zn_28_ alloy. This means that the rearrangement of atoms present during the glass transition process requires more energy than for the crystal nucleation and growth process. In turn, the activation energy calculated for the beginning of α-Mg phase crystallization is higher than for the maximum of the peak crystallization of the α-Mg phase and even higher than for the maximum of the peak crystallization of the Mg_12_Zn_13_ phase, which indicates that the value of activation energy decreases during the crystallization process. In conclusion, it can be stated that the crystallization process is a lower energy barrier in relation to the glass transition process. Therefore, the crystallization process has a higher probability of occurrence. In addition, there is a direct relationship between the activation energy and the reaction rate: the lower the activation energy, the faster the reaction rate.

## 4. Conclusions

X-ray diffraction studies revealed the presence of the α-Mg phase and the intermetallic Mg_12_Zn_13_ phase appearing during isothermal annealing.

The increase in the isothermal annealing temperature from 352 K to 355 K practically does not affect the change in incubation time. In addition, the α-Mg phase nucleates earlier than the Mg_12_Zn_13_ phase.

In the temperature range from 475 K to 599 K, the disappearance of the Mg_12_Zn_13_ phase was observed in favor of the Mg_2_Zn_11_ phase, with a simultaneous rise of the contribution of α-Mg.

Activation energies for three temperature values, i.e., glass transition, onset, and crystallization peak, were calculated by the Kissinger method, and their values were: *E*_g_ = 176.91, *E*_x_ = 124.26, *E*_p1_ = 117.49 and *E*_p2_ = 114.48 kJ mol^−1^, respectively.

The smallest energy barrier is shown by the crystallization process in relation to the glass transition process. This means that the crystallization process has a greater chance of occurring in the structure under normal conditions. In addition, as the transformation progresses, the activation energy decreases. Since the system tends to an energy minimum, the crystal structure is a stable structure.

It has been proven that selecting the appropriate heat treatment procedure (temperature-time) will allow for obtaining a crystalline-amorphous material with the assumed degree of crystallinity, in which the amorphous phase is the matrix, and the reinforcing phase is the crystalline phase.

## Figures and Tables

**Table 1:** Characteristic temperatures: *T*_g_, *T*_x_, *T*_p,_ and undercooling degree Δ*T*_x_ for amorphous Mg_72_Zn_28_ alloy heated at different rates.

Heating Rate *β* (K/min)	*T*_g_ (K)	*T*_x_ (K)	*T*_p1_ (K)	*T*_p2_ (K)	Δ*T*_x_ *= T*_x_ − *T*_g_ (K)
5	-	356.3	371.0	382.6	-
10	-	363.0	378.0	388.5	-
20	360.6	368.0	383.0	395.8	7.4
40	364.0	372.6	389.0	403.5	8.6
80	368.9	381.2	401.0	412.5	12.3

## Data Availability

Not applicable.
